# Extra-hippocampal contributions to pattern separation

**DOI:** 10.7554/eLife.82250

**Published:** 2023-03-27

**Authors:** Tarek Amer, Lila Davachi

**Affiliations:** 1 Department of Psychology, University of Victoria Victoria Canada; 2 Department of Psychology, Columbia University New York United States; 3 Nathan Kline Research Institute Orangeburg United States; University of Pennsylvania United States; National Institute of Mental Health United States

**Keywords:** pattern separation, hippocampus, prefrontal cortex, cognitive control

## Abstract

Pattern separation, or the process by which highly similar stimuli or experiences in memory are represented by non-overlapping neural ensembles, has typically been ascribed to processes supported by the hippocampus. Converging evidence from a wide range of studies, however, suggests that pattern separation is a multistage process supported by a network of brain regions. Based on this evidence, considered together with related findings from the interference resolution literature, we propose the ‘cortico-hippocampal pattern separation’ (CHiPS) framework, which asserts that brain regions involved in cognitive control play a significant role in pattern separation. Particularly, these regions may contribute to pattern separation by (1) resolving interference in sensory regions that project to the hippocampus, thus regulating its cortical input, or (2) directly modulating hippocampal processes in accordance with task demands. Considering recent interest in how hippocampal operations are modulated by goal states likely represented and regulated by extra-hippocampal regions, we argue that pattern separation is similarly supported by neocortical–hippocampal interactions.

## Introduction

Repetition of common activities within our daily lives results in highly similar and overlapping experiences. To distinguish between these experiences in memory, unique traces are formed for each experience based on the orthogonalization of overlapping features – a process referred to as ‘pattern separation’. The presence of overlapping features in two stimuli or events creates an overlap in resulting activity patterns, and thus, a redundancy between input patterns. If this redundancy is not reduced, a novel, but similar, experienced event can result in major interference and reactivation of a previously stored memory. Pattern separation reduces this redundancy by transforming similar input patterns into less similar output patterns, ultimately allowing distinct memories for overlapping events to be stored (O’Reilly and McClelland, 1994). Thus, the need to transform or differentiate input patterns to prevent interference from and reactivation of overlapping stimuli/memories defines pattern separation function. Behaviorally, this typically involves the successful discrimination of lure stimuli from targets with highly overlapping features (see Zotow et al., 2020 for an alternative method to measure pattern separation behaviorally based on the conditional probability of retrieving similar memories). It is important to note here that although the term ‘pattern separation’ has its roots in computational neuroscience, it has been used widely by different researchers to describe a range of processes (e.g., computational processes or behavioral discrimination), arguably degrading its construct validity (see Santoro, 2013). Our use of the term in the current paper (which we believe is important as it has been used in the studies we reference) focuses on the neural processes presumed to be necessary to perform behavioral discrimination and, in most cases noted, refers to the increased distinctiveness in evoked neural patterns to stimuli that are perceptually overlapping. Although the use of the term commonly refers to these behavioral/mnemonic discrimination processes, it should be highlighted that behavioral discrimination incorporates multiple cognitive processes, with pattern separation being only one. Task demands typically dictate the extent of pattern separation involvement (or differentiation of similar inputs), and whether it is truly isolated from other mnemonic and perceptual processes involved in behavioral discrimination. Thus, the proper usage of the term ‘pattern separation’ and the validity of the tasks that aim to measure it are topics of ongoing debate. Nonetheless, to summarize, the neural and behavioral discrimination of overlapping activity patterns and stimuli, respectively, reflecting successful interference resolution, define pattern separation in the current paper. While other memory processes, such as recognition and source memory, may recruit similar brain-wide regions, interactions, or mechanisms, pattern separation (which can be involved in these memory processes depending on the conditions) requires the need to resolve interference from an overlap in stimulus features and evoked neural responses (e.g., see Stevenson et al., 2020 for evidence of distinct neural mechanisms associated with pattern separation and source memory).

A large body of work, across methods, analytical techniques, and different species, has focused on hippocampal contributions to pattern separation (e.g., O’Reilly and McClelland, 1994; Treves and Rolls, 1994; Leutgeb et al., 2007; Yassa and Stark, 2011b; LaRocque et al., 2013; Neunuebel and Knierim, 2014; Kyle et al., 2015; Berron et al., 2016; Sakon and Suzuki, 2019). This research has provided convincing evidence for reduced representational overlap for more similar events or stimuli within the hippocampus compared to events with less featural overlap. These findings are consistent with theorizing that the hippocampus contains neural circuitry that promotes the allocation of dissimilar neural populations to highly similar events, thus facilitating the mnemonic discrimination of similar experiences through their reduced neural overlap. However, a careful examination of the broader literatures on episodic memory, attention, interference resolution, and perception suggests that the process of pattern separation may be a more widespread and iterative process supported by a *network of brain regions*. Here, we review evidence that patterns of neural activity occurring in cognitive control and sensory regions (1) are sensitive to pattern separation demands and show hippocampal-like activity in response to overlapping stimuli, (2) temporally precede pattern separation activity in the hippocampus, and thus filter or ‘separate’ the signal reaching the hippocampus, likely influencing hippocampal output, and (3) are correlated with memory performance on tasks that rely on pattern separation, demonstrating their functional relevance. Accordingly, we highlight that pattern separation is best considered the result of network interactions and not one solely supported by intra-hippocampal processes. While previous studies have not explicitly argued that pattern separation occurs solely in the hippocampus, a focus on hippocampal processes (stemming from animal electrophysiological studies) due to their potentially unique contribution in performing an extreme version of pattern separation (see ‘A unique hippocampal role in pattern separation?’), has shifted attention away from the important domain-general contribution of extra-hippocampal regions. Although this is beginning to change (e.g., Johnson et al., 2021; Nash et al., 2021), a unifying framework has yet to be put forward. We contend that a full understanding of mnemonic discrimination requires such a framework promoting an understanding of how the hippocampus interacts with and is modulated by input from brain-wide regions during pattern separation. In particular, based on the reviewed evidence, we argue that task goals, supported by cognitive control regions, may mediate pattern separation in sensory regions feeding into hippocampus (thus regulating its cortical input), or more directly target the hippocampus, biasing it toward pattern separation. Although we will discuss cognitive control and sensory function in the context of findings demonstrating activity in typically lateral frontal and occipito-temporal regions, respectively, we acknowledge the complexity of brain-cognition mappings and the dynamic brain network properties underlying cognitive functions outlined by data-driven approaches (e.g., Cocuzza et al., 2020; Keane et al., 2021; Klein, 2012; Poldrack and Yarkoni, 2016). The aim of the proposal is not to stress a potential role for these particular regions in pattern separation, but to more generally highlight extra-hippocampal contribution to the overall process, and similar to data-driven approaches, underscore the importance of widespread brain networks for a cognitive process. Finally, in light of the continued interest in the many functions of the hippocampus and recent focus on how its activity patterns reflect top-down goals (e.g., Aly and Turk-Browne, 2018; Aoki et al., 2019; Haimerl et al., 2019), it is important now more than ever to carefully examine how and when other brain regions might directly support pattern separation or coordinate and modulate hippocampal contributions to pattern separation.

### Hippocampal contributions to pattern separation

#### Animal studies

Considering the structural complexity of the hippocampus and its widespread contributions to memory, much theoretical and experimental work has focused on developing an understanding of how hippocampal subfields and their inter-connectivity might support distinct memory functions such as pattern separation and completion (O’Reilly and McClelland, 1994; Rolls, 2013). This research has typically, but not exclusively, focused on the rodent hippocampus through simultaneous electrophysiological recordings from different hippocampal subfields while animals explore and navigate within different controlled environments. For example, several studies recorded activity from the dentate gyrus (DG) and area CA3 of the hippocampus while the testing environment was incrementally manipulated by varying degrees (i.e., small changes were gradually incorporated into an original, well-learned environment; Leutgeb et al., 2007; Neunuebel and Knierim, 2014; Knierim and Neunuebel, 2016). These studies demonstrated that the DG, in particular, showed large changes in neural *firing patterns* or *rates* (‘global’ or ‘rate remapping’) in response to minimal changes in the environment, providing support for the notion that DG neural processing promotes pattern separation. Specifically, evidence from these studies suggested that the DG engages in pattern separation by amplifying small changes in its cortical input, allowing it to maintain separated representations or assign distinct neuronal codes to similar events (see Sakon and Suzuki, 2019 for similar evidence in monkeys). Input to the DG relies on the perforant path, which connects the DG to entorhinal cortical neurons, and supports pattern separation, considering that degraded perforant input to the DG in both animals and humans has been linked to pattern separation deficits (e.g., Wilson et al., 2006; Yassa et al., 2011a; Burke et al., 2018). The process of assigning distinct neuronal codes to overlapping inputs in the DG is thought to be accomplished by sparse coding, in which both the proportion of active neurons and the mean firing rate for a single event are low, providing the DG with the capacity to distinctly represent a large number of events with highly overlapping features (Marr, 1971; Treves and Rolls, 1994; Hunsaker and Kesner, 2013; Piatti et al., 2013). This consequently minimizes interference from similar inputs and facilitates the storage of detail-specific, non-overlapping memories. In particular, the signal projected forward from the DG to CA3 is sufficiently sparse such that similar inputs from overlapping events are unlikely to activate the same set of neurons in CA3 – a subregion with an auto-associative function that ‘pattern completes’ partial or degraded input based on previously stored representations (O’Reilly and McClelland, 1994). This process is thought to be additionally facilitated by sparse mossy fiber connectivity between the DG and CA3, setting the ‘sparseness’ in CA3 or forcing pattern separated representations onto CA3 in response to overlapping stimuli (Rolls, 2007; Rolls, 2013; see also Lassalle et al., 2000). The activation of different CA3 neural sets thus prevents automatic pattern completion from overlapping inputs (and recall of a previously stored memory), thus allowing the storage of distinct memories (i.e., similar inputs are transformed into less similar or divergent outputs).

Although the discussed work suggests a critical role for the hippocampus in pattern separation of similar spatial environments or contexts during perception (i.e., when an animal encounters a similar environment with no memory demands) (see Rolls, 1996), other work has emphasized the importance of the hippocampus for non-spatial pattern separation with mnemonic demands. Specifically, this work highlights the contribution of the hippocampus when a mnemonic load, such as the need to recall similar representations over long delays, is present in a pattern separation task. For example, one study in rats demonstrated that in an odor discrimination delayed match-to-sample task, the DG is critical for olfactory pattern separation only during long delays between the sample and test phases (Weeden et al., 2014). In particular, rats with DG lesions were required to discriminate between odors that varied in their similarity at short (15 s) or long (60 s) delays. The findings showed that although rats with DG lesions performed similar to controls in the short delay condition (demonstrating intact perceptual pattern separation and potential involvement of extra-hippocampal regions in similar odor discrimination), they showed significant impairments in the long delay condition, particularly for highly similar odors requiring some form of pattern separation. Consistent with previous work, this finding suggests that the hippocampus is critical for pattern separation, but especially when similar odor representations needed to be recalled during longer delays between the sample and test phases. Taken together, animal studies suggest that hippocampal subfields and connections are ideally structured to support pattern separation of spatial contexts and non-spatial stimuli, particularly when they include mnemonic demands.

### Human studies

Although animal work has typically focused on the dorsal hippocampus and manipulated spatial features, neuroimaging work in humans has allowed examination of hippocampal subfields, along with the entire brain, while also using paradigms where similarity could be manipulated for objects and their features. This is particularly important as it has provided a more complete view of pattern separation functions of the hippocampal formation as they relate to complex human behaviors (e.g., mnemonic discrimination). The first study to report evidence for perceptual pattern separation in the human hippocampus used a functional magnetic resonance imaging (fMRI) adaptation paradigm during an incidental encoding memory task, known as the Mnemonic Similarity Task (incidental version with no explicit memory demands). The study showed that during performance of an ‘indoor’/‘outdoor’ judgment task on individual items, the magnitude of blood oxygenation level-dependent (BOLD) activation in a region encompassing human DG and area CA3 (lower spatial resolution in earlier fMRI studies did not allow isolation of these two subfields) was statistically indistinguishable for completely novel items and similar lures (i.e., images of novel items that are highly similar to previously presented items) (Bakker et al., 2008). That is, unlike other regions within the medial temporal lobe (MTL), DG/CA3 showed repetition suppression for identical repeats, but not lure items, suggesting that it was treating the lure items as new (see Berron et al., 2016 for evidence based on ultra-high-resolution fMRI that this effect is localized to the human DG).

**Figure 1.**
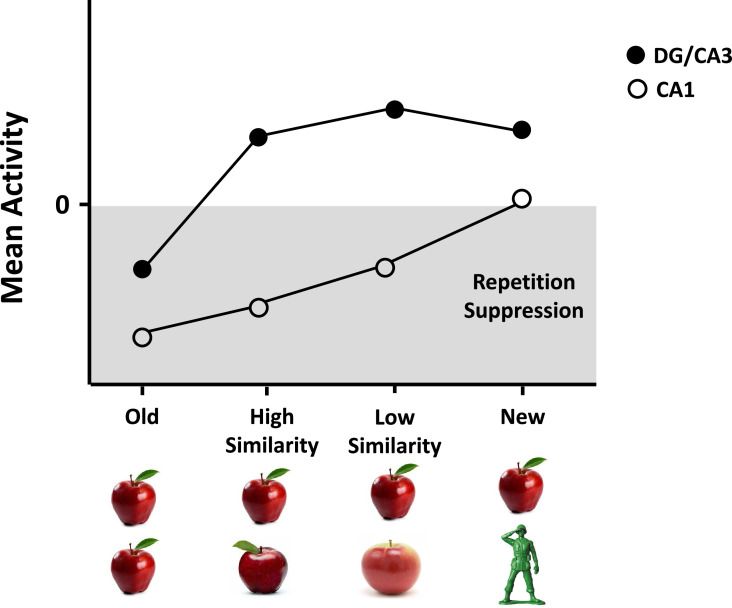
Pattern separation in dentate gyrus (DG)/CA3 in human studies. Relative to activity levels in response to repetitions of identical items in an incidental encoding memory task, DG/CA3, but not CA1, shows a similar, rapid nonlinear increase in activity level in response to lures with high similarity, lures with low similarity, and new items. This activation profile is best fit with a power function that has been used to characterize pattern separation in brain regions. Figure adapted from Figure 4 of Yassa and Stark, 2011b.

Follow-up studies manipulated the degree of similarity between lure and old items and demonstrated again that DG/CA3 showed evidence of pattern separation for highly similar lures, consistent with animal studies showing that the DG responds to minimal environmental changes (Lacy et al., 2011; Yassa et al., 2011a). In particular, relative to the reduced activity in response to identical repeats (i.e., repetition suppression), DG/CA3, but not other subfields such as CA1, showed increased activity for (1) lures with high similarity, (2) lures with low similarity, and (3) new items. This increased activity was at a similar level for all three item types, such that the activation profile was best fit with a power function when input (extent of change from old item) and output (change in activation level) are mapped (see Figure 1). This power function has since been used in fMRI studies to characterize pattern separation (e.g., Motley and Kirwan, 2012).

In addition to univariate analyses that only assess brain activation levels (similar to studies reporting rate remapping), recent studies have used multivariate techniques to examine the representational content in the hippocampus when presented with similar stimuli. These studies have demonstrated that, compared to extra-hippocampal or other MTL regions, the hippocampus shows *lower representational overlap* (i.e., distinct activity patterns) between stimuli with overlapping features, providing evidence for orthogonalized representations theorized to emerge from pattern separation (LaRocque et al., 2013; Hulbert and Norman, 2015; Kyle et al., 2015; Favila et al., 2016; Chanales et al., 2017). For example, one study demonstrated that the similarity between activation patterns for similar scenes encountered in an associative memory paradigm was reduced in the hippocampus relative to extra-hippocampal regions (Favila et al., 2016). Critically, the extent of this reduced similarity in activation patterns in the hippocampus (i.e., pattern separation) was associated with future memory and reduced behavioral interference between the scenes (see also Wanjia et al., 2021). Another study with high spatial resolution illustrated a similar effect for learned spatial environments particularly in the DG/CA3 area, consistent with the findings reported in univariate studies (Kyle et al., 2015).

Finally, in accordance with the imaging findings, several neuropsychological studies have demonstrated that patients with hippocampal damage show deficits in behavioral indices of mnemonic pattern separation, as indexed by false recognition of lures on the standard version of the Mnemonic Similarity Task, in which participants identify if presented objects are ‘old’, ‘similar’, or ‘new’ (Motley and Kirwan, 2012; Baker et al., 2016; Hanert et al., 2019a; Hanert et al., 2019b). For example, one study demonstrated that a patient with a rare, selective lesion to the DG showed similar performance to healthy control participants in identifying target items and foils but was severely impaired in correctly rejecting lure items (Baker et al., 2016). Taken together, these findings provide convincing evidence that the hippocampus, and particularly hippocampal subfield DG, supports pattern separation by assigning distinct neural codes to overlapping events, thus reducing interference and representational similarity between these events (but see Quian Quiroga, 2020 for an alternative viewpoint).

### Extra-hippocampal contributions to pattern separation

#### Attention and interference control

Although the literature has typically focused on the role of the hippocampus in pattern separation, the contribution of processes in extra-hippocampal regions has received less attention. However, a distinct but substantial literature has illustrated that regions in the frontoparietal control network contribute to functions similar to pattern separation (or result in outcomes that are consistent with pattern separation function), such as resolving interference between competing memories or stimuli (e.g., Badre and Wagner, 2007; Amer et al., 2016). This raises important questions about whether and how these processes directly contribute to, or modulate, hippocampal pattern separation. Given their robust activation across several tasks and contexts involving conflict, frontoparietal regions have been hypothesized to play a domain-general role in interference resolution. For example, early neuroimaging studies show that regions, such as the ventrolateral prefrontal cortex (VLPFC; a region typically associated with cognitive control), are activated in working memory tasks that require resolving interference from related, but previously relevant, information (e.g., Jonides et al., 1998; Jonides et al., 2000; Öztekin et al., 2009). Particularly, in paradigms that require identifying whether a probe item is part of a previously presented set of target stimuli, the VLPFC shows greater activity on high interference trials in which the probe does not match one of the relevant targets, but instead matches one of the targets from the immediately preceding, irrelevant trial. Thus, overcoming an interfering, familiar stimulus – a primary demand in pattern separation tasks – engages top-down control or attention-related regions.

In addition to working memory tasks, similar control-mediated interference resolution mechanisms have been reported during episodic memory retrieval (see Badre and Wagner, 2007 for a review). For example, in associative memory tasks in which a word is paired with two images from different categories (e.g., faces and scenes) during encoding, retrieval of an image from only one of the categories (such that the image from the irrelevant category causes interference), engages lateral frontal regions to resolve retrieval competition (Kuhl et al., 2011). Additionally, the extent of this frontal engagement has been associated with suppression of the irrelevant image and improved memory performance, illustrating the behavioral relevance of this frontal activity (Wimber et al., 2015). These findings are consistent with activity patterns reported in classic retrieval induced forgetting paradigms, wherein subjects engage in retrieval practice of a subset of items at the expense of unpracticed, related items (Kuhl et al., 2007; Wimber et al., 2009; see also Penolazzi et al., 2014), and are also consistent with frontal engagement reported in source memory tasks that require retrieving a memory-specific context while resolving interference from similar, competing contexts (Rugg et al., 1999; Dobbins et al., 2002; King et al., 2005). Collectively, these studies illustrate that, similar to hippocampal pattern separation, cognitive control processes contribute to memory specificity and success by resolving competition between interfering stimuli or memory representations.

### Mechanisms of interference control

One mechanism that has been proposed to support control-mediated interference resolution is the top-down modulation of information processing in sensory regions that process incoming stimuli. Neural responses in sensory regions are typically enhanced or suppressed based on whether task-relevant or irrelevant information is being processed. For example, several studies have illustrated that relative to baseline (passive) viewing conditions, instructions to attend to or ignore images of scenes, respectively, result in upregulation or downregulation of activity in the scene-selective ‘parahippocampal place area’ (Gazzaley et al., 2005; Rissman et al., 2009). Functional connectivity analyses have further revealed that these modulatory signals originate in top-down control regions, such as the lateral and anterior PFC (Kastner and Ungerleider, 2001; Miller and D’Esposito, 2005; Gazzaley et al., 2007; see Baldauf and Desimone, 2014 for such evidence based on oscillatory analyses with high temporal resolution). Similarly, there is evidence that, during memory retrieval, competition between retrieved items modulates activity in sensory regions that feed into the hippocampus (Kuhl et al., 2011; Wimber et al., 2015). For example, in an associative memory task, suppression of competitors has been shown to occur particularly in category-selective regions of the ventral visual cortex, and critically, to be correlated with the level of lateral frontal activation during retrieval (Wimber et al., 2015). This provides an example of how extra-hippocampal processes in frontal and visual cortical regions contribute to memory functions (e.g., associative memory) typically ascribed to the hippocampus (see also Dennis et al., 2012; Wing et al., 2015).

Given that changes in information processing in these top-down regulated sensory regions should feedforward into the hippocampus, the discussed findings raise the interesting possibility that cognitive control or attention mechanisms contribute to hippocampal pattern separation functions by regulating its cortical input, thus influencing hippocampal output due to altered input. This proposal is also consistent with network models of memory more broadly, which suggest that memory function is an emergent property of interactions between networks of brain regions, and in particular, neocortical–hippocampal interactions. For example, several studies have shown that, during successful memory formation, the hippocampus shows connectivity with an extensive network of brain regions, including lateral frontal and temporal, medial parietal, and occipital regions, which presumably provide cortical input to the hippocampus and regulate its function (e.g., Ranganath et al., 2005; Schott et al., 2013; Keerativittayayut et al., 2018). Based on these findings, memory network models have emphasized the role of the hippocampus as a convergence zone, integrating information from multiple brain regions and flexibly weighting cortical input based on top-down modulation and task goals (Mišić et al., 2014; Backus et al., 2016; Aly and Turk-Browne, 2018). Thus, considering the described evidence, we propose the ‘cortico-hippocampal pattern separation’ (CHiPS; ‘Hi’ = hippocampal) framework that accounts for pattern separation contributions from different regions throughout the brain, and we discuss evidence supporting this framework in the following sections. In particular, we first discuss neural and lesion evidence from human studies and interactions between brain regions that support pattern separation, then we discuss relevant animal work, and finally, consider a potential unique role of the hippocampus in pattern separation.

### Pattern separated input to the hippocampus

#### Neural evidence

Thus far, we have discussed the potential contributions of extra-hippocampal regions to pattern separation by reviewing evidence that these regions are engaged in related processes, such as interference resolution. Extending this work, recent studies have provided more direct evidence of the contribution of these regions to pattern separation using tasks that are particularly designed to assess pattern separation (but see Zotow et al., 2020 for limitations and questions regarding the validity of these tasks). For example, using the previously discussed Mnemonic Similarity Task, in which activation elicited in response to repeated and lure items is compared in an incidental encoding task, one study demonstrated that both frontal and sensory regions show hippocampal-like univariate activity patterns in response to lure items (Pidgeon and Morcom, 2016). In particular, lateral frontal and occipito-temporal cortical regions showed elevated activity (or no repetition suppression) in response to lure (and new), but not repeated, items, consistent with a perceptual pattern separation process in these regions. Additionally, curve fitting analyses illustrated that activity in the same frontal regions can be fit with a power function in response to graded changes in the similarity of the lures (i.e., high to low similarity), in line with what has previously been shown for DG/CA3 in the hippocampus (e.g., Lacy et al., 2011). Interestingly, these findings providing evidence for extra-hippocampal perceptual pattern separation are consistent with foundational perceptual studies demonstrating that sensory occipital regions do not exhibit repetition suppression, and are thus sensitive to, minimal changes in the physical properties or features of previously presented stimuli (e.g., Koutstaal et al., 2001; Rotshtein et al., 2005; Kim et al., 2009). In addition to the perceptual work, other studies have provided evidence of extra-hippocampal pattern separation in the mnemonic domain. For example, a recent study illustrated that a set of frontal and visual regions showed elevated activity when correctly rejecting (compared to falsely recognizing) lures in the test phase of the Mnemonic Similarity Task, suggesting sensitivity to pattern separation demands in these regions (Nash et al., 2021; see also Klippenstein et al., 2020). Similarly, top-down expectations, typically associated with frontal mechanisms, have been shown to influence the extent of hippocampal pattern separation of highly similar objects in a recognition task (Frank et al., 2020). Furthermore, studies have provided corroborating evidence for pattern separation capacities outside the hippocampus by using multivariate analyses to demonstrate that activity patterns for similar, lure stimuli in a memory task can be distinguished from previously presented stimuli in both frontal and occipital regions (Bowman et al., 2019). Finally, a recent study demonstrated the potential role of extra-hippocampal regions in pattern separation by illustrating that resting-state functional connectivity patterns in broad cortical networks, including frontal, temporal, and hippocampal regions, were associated with behavioral performance on the Mnemonic Similarity Task (Wahlheim et al., 2022; see also Bennett and Stark, 2016 for evidence that mnemonic discrimination is associated with the integrity of various white matter tracts that provide cortical input into the MTL and hippocampus).

In addition to frontal and occipito-temporal regions, evidence suggests that extra-hippocampal regions within the MTL contribute to pattern separation. In particular, regions such as the parahippocampal cortex (PhC), perirhinal cortex (PrC), and entorhinal cortex (ErC; comprised of the posteromedial; pmErC; and anterolateral; alErC; subregions), are thought to play a role in resolving object and spatial interference from overlapping stimuli to promote stronger hippocampal pattern separation. Multiple studies have illustrated that these regions show increased activity when discriminating object and spatial lures from targets (i.e., no repetition suppression), with the PrC and alErC typically showing selectivity for object interference (or object processing, in general), and the PhC and pmErC, while less robust, showing selectivity for spatial interference (Reagh and Yassa, 2014; Berron et al., 2018; Reagh et al., 2018). With respect to object processing, in particular, a growing body of work using various tasks and methodologies has provided evidence that the PrC is critical for perceptual and mnemonic discrimination of objects, given its role in representing objects in their most ‘integrated’ form. That is, considering that the PrC (and the alErC that it projects to) form the apex of the processing hierarchy of the ventral visual stream, object features, which progressively integrate along the stream, reach their final integrated form in these MTL regions based on the conjunction of complex visual and conceptual features (Murray and Bussey, 1999; Barense et al., 2007; Kent et al., 2016). This consequently promotes the differentiation and discrimination of overlapping objects, as evidenced, for example, by PrC activity patterns uniquely representing individual objects based on their integrated features (e.g., Erez et al., 2016; Martin et al., 2018) and differentiating highly similar objects within categories with highest precision in the ventral visual stream (Ferko et al., 2022; see also O’Neil et al., 2009 for similar conclusions based on univariate analysis). In sum, such evidence of activity and connectivity patterns consistent with pattern separation in regions upstream of the hippocampus (both within and outside of the MTL) is consistent with the CHiPS framework, wherein hippocampal input is in some cases already filtered or ‘separated’ to an extent, potentially altering subsequent hippocampal output on the already-processed input.

Thus far, we have reviewed empirical evidence showing that regions outside of the hippocampus are indeed sensitive to overlapping stimulus features and that their engagement during tasks contributes to behavioral discrimination. However, a critical question and element of the ChiPS framework is that extra-hippocampal regions are processing old (repeated) items and similar lures differently *before* this cascade of neural activity is processed in the hippocampal formation. In order to better understand the relationship between pattern separation in the hippocampus and in regions directly feeding into it, one can turn to studies that provide temporal information about *when* pattern separated responses emerge in these distinct brain regions. A recent electrocorticography (ECoG) study demonstrated that electrophysiological signatures of pattern separation in the occipito-temporal cortex *temporally precede* those in the hippocampus (Lohnas et al., 2018). In a continuous recognition paradigm, both the occipito-temporal cortex and hippocampus distinguished between old and lure items, as indexed by differences in univariate high-frequency activity to correctly identified items. Critically, this effect occurred up to 500 ms earlier in the occipito-temporal cortex, providing strong evidence that extra-hippocampal pattern separation occurs prior to, and thus is not induced by, the hippocampus. Another recent study illustrated that the relationship between hippocampal BOLD activity and performance on a pattern separation task is impacted by processes in the lateral occipital cortex (Koolschijn et al., 2019). In particular, consistent with previous studies, activity in the hippocampus predicted behavioral memory interference in a task that required participants to learn context-specific shape associations. However, this hippocampal–behavioral relationship disappeared when greater interference (measured both neurally and behaviorally) was induced in the lateral occipital cortex using transcranial direct current stimulation. Finally, an aging study provided similar evidence by demonstrating that well-documented pattern separation deficits in older adults can, at least partly, be accounted for by representations maintained in regions that feed into the hippocampus (Weeks et al., 2020). Specifically, in a delayed match-to-sample task that required participants to maintain in memory only a subset of presented (relevant) images for a subsequent old-lure discrimination task, older adults with reduced cognitive control abilities (e.g., Amer et al., 2016), maintained information from irrelevant images in MTL regions (including the hippocampus) and in regions that feed into the hippocampus (including the lateral occipital cortex), relative to young adults. Importantly, the extent to which this irrelevant information was maintained was associated with behavioral performance on the mnemonic discrimination task across both age groups. Taken together, these findings suggest that the hippocampus is involved in maximally separating overlapping stimuli (e.g., items with similar features or same items in different contexts), however, these processes may leverage already separated (or less noisy) representations that propagate into the hippocampus from upstream sensory and MTL regions.

### Lesion studies

Although patients with hippocampal lesions show evidence of intact recognition memory, in which they can discriminate old items from new (foil) items with no featural overlap (a task that presumably does not require pattern separation; e.g., Vargha-Khadem et al., 1997; Bastin et al., 2004; Holdstock et al., 2008), other work has indicated that they make more false alarms to similar lures relative to healthy control participants on the Mnemonic Similarity Task (e.g., Motley and Kirwan, 2012; Baker et al., 2016). These latter findings illustrate the importance of the hippocampus (particularly the DG) to pattern separation and suggest that it is critical for the discrimination of overlapping stimuli/events, which drives memory specificity and success. Interestingly, research on patients with frontal lesions has similarly demonstrated a significantly greater vulnerability to lure false alarms (Curran et al., 1997; Delbecq-Derouesné et al., 1990; Rapcsak et al., 1999; Schacter et al., 1996; for a review see Festini and Katz, 2021), particularly when those lures show high feature overlap with their respective targets (Parkin et al., 1996), or when detail-oriented recollective processes are required to reject and overcome familiarity of the lures (Thompson-Schill et al., 2002; Verfaellie et al., 2004). These findings are further supported by recent work demonstrating that, in older adults with typical deficits in frontal or control function, measures of frontally mediated cognitive control are associated with lure discrimination on the Mnemonic Similarity Task (Foster and Giovanello, 2020; Gellersen et al., 2021; Pishdadian et al., 2020). Similarly, impairments in cognitive control/executive function have been shown to be associated with mnemonic discrimination deficits in older adults at risk for mild cognitive impairment, further illustrating a functional link between cognitive control and pattern separation (Gellersen et al., 2023).

In addition to the patient work on mnemonic discrimination, lesion studies using perceptual discrimination tasks (i.e., tasks requiring pattern separation with no long-term memory demands) further illustrate that pattern separation is supported by a network of brain regions. For example, several studies have demonstrated that although the hippocampus seems to be critical for spatial perception and discrimination (e.g., Behrmann et al., 2016; Lee et al., 2012), patients with hippocampal lesions can discriminate between similar faces, objects, and paintings, suggesting intact pattern separation for different stimuli in the perceptual domain (e.g., Lee et al., 2005; Ruiz et al., 2020). Unlike hippocampal lesions, lesions in upstream MTL regions, particularly the PrC, seem to negatively impact perceptual discrimination. For example, relative to controls, patients with damage to the PrC show impaired performance when discriminating both familiar and unfamiliar objects with high, but not low, featural overlap in perceptual discrimination tasks (e.g., Barense et al., 2005; Barense et al., 2007; Barense et al., 2010; but see Mitchnick et al., 2022 for recent evidence that the DG may also be involved in the perceptual discrimination of similar objects). In sum, the lesion findings collectively provide support for the hypothesis that pattern separation, with or without long-term memory demands, relies on a network of brain regions that discriminate and resolve interference between similar or competing stimuli/memories, ultimately storing distinct, non-overlapping memories.

### Region interactions to support pattern separation

**Figure 2.**
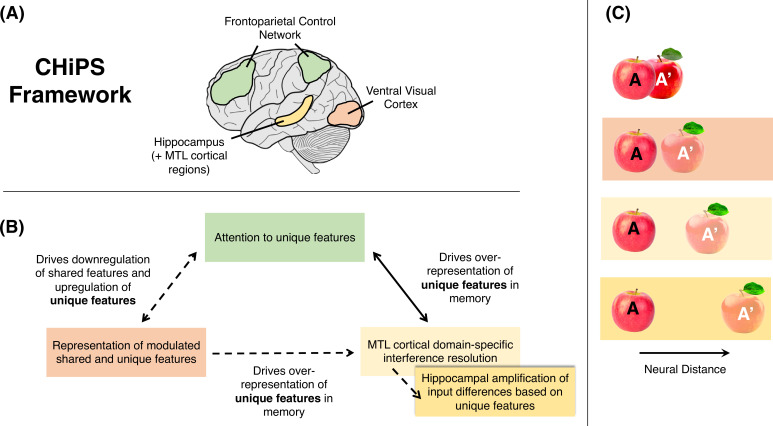
Schematic of proposed cortico-hippocampal pattern separation (CHiPS) framework. (**A**) We propose that pattern separation is supported by input from a network of brain regions and initiated outside the hippocampus in frontoparietal control, sensory/visual, and medial temporal lobe (MTL) cortical regions. (**B**) In particular, in one pathway (dotted arrows) cognitive control regions modulate sensory regions (after receiving early novelty signal feedback from sensory and hippocampal regions), such that unique, non-overlapping features in similar stimuli are upregulated while overlapping features are downregulated. This drives an increase in neural representation of unique features in sensory regions, creating different (or ‘separated’) representations of similar stimuli and regulating MTL cortical input. Once this signal reaches extra-hippocampal MTL cortices, such as the perirhinal cortex, domain-selective (i.e., object vs. spatial) interference resolution based on modulated features takes place before the signal reaches the hippocampus. Based on this filtered input, the hippocampus maximizes (or amplifies) the outlined feature differences in a domain-general fashion and forms unique memory traces for overlapping stimuli with more differentiated representations (i.e., the filtered input to the hippocampus drives over-representation of unique features in memory). An alternative, but not mutually exclusive, possibility (solid arrow) is that cognitive control regions directly modulate the hippocampus and its representations to enhance pattern separation in line with task demands. (**C**) Using apple A and apple A’ as an example of two similar inputs (where A was initially viewed and stored in memory), control regions drive separation of the two inputs in sensory regions by upregulating unique features (e.g., the leaf) and downregulating overlapping features in A’. The separation (or neural distance) of the two inputs is further increased in MTL cortical regions and then the hippocampus, creating a unique memory trace for each apple.

Given evidence that hippocampal and extra-hippocampal regions are involved in dissociating overlapping stimuli, how do interactions between these different regions overall support pattern separation as proposed by the CHiPS framework? Based on work illustrating control-mediated, top-down regulation of sensory regions feeding into the hippocampus, and that the hippocampus acts as a convergence zone integrating information from multiple brain regions during memory function (e.g., Backus et al., 2016; Mišić et al., 2014), we hypothesize that frontoparietal control areas facilitate pattern separation by resolving interference from overlapping stimuli in early sensory regions, thus regulating hippocampal input. This facilitation can occur when viewing an overlapping stimulus with no explicit long-term memory demands (perceptual pattern separation resulting in the formation of a new memory), or in pattern separation tasks that demand explicit memory decisions in response to comparison to a stored memory representation (i.e., cognitive control function resolves interference and aids in the precise discrimination of overlapping activity patterns elicited by sensory input and stored representations). Following additional interference resolution in upstream MTL regions (e.g., PrC and alErC), the hippocampus, and particularly the DG, consequently forms unique memory traces of the overlapping stimuli, as indicated by prior work (e.g., Yassa and Stark, 2011b), by maximizing outlined feature differences between those stimuli to distinguish between them in memory (see Figure 2 for a schematic of the proposed CHiPS framework). Specifically, through sparse coding in the DG which links input patterns in the ErC to non-overlapping sets of neurons in CA3, the hippocampus further differentiates input patterns from overlapping stimuli/representations, allowing the storage and subsequent recall of distinct memories (e.g., Norman and O’Reilly, 2003). The degree to which this hippocampal process successfully results in the storage of non-overlapping memories should rely to an extent on the pre-existing differentiation of input patterns to the ErC driven by extra-hippocampal regions. Fully accounting for the state of MTL input, in addition to overall neocortical–hippocampal interactions, should strengthen computational frameworks of pattern separation. Thus, according to the CHiPS framework, the processes enacted by extra-hippocampal and hippocampal regions comprise a continuum in resolving interference, with the DG playing a particular role in amplifying feature differences in memory traces (e.g., see Reagh and Yassa, 2014; Lohnas et al., 2018 for evidence that the DG/hippocampus may play a unique role in influencing behavioral or mnemonic decisions in pattern separation tasks). This unique DG role might potentially reflect a ‘break point’ in the pattern separation process for consolidated memories, with memories consolidated through DG containing amplified featural differences (see ‘A unique hippocampal role in pattern separation?’).

Consistent with the continuum view of resolving interference, there is evidence from visual search and selective attention tasks that frontoparietal regions encode simple feature properties and bias downstream sensory regions to represent those features based on attentional demands (e.g., Ester et al., 2016; Bichot et al., 2019). Such feature-based attention can thus initiate pattern separation by driving attention toward diagnostic features that distinguish between similar events. The mechanism by which this occurs may involve upregulation of unique features across stimuli and simultaneous downregulation of overlapping features (see Moher et al., 2014 for evidence of goal-directed inhibition of distractor features during early visual processing). Although frontoparietal regions, including lateral frontal, intraparietal sulcus, and superior parietal regions, are the likely source of this feature modulation (e.g., Ester et al., 2016; Pidgeon and Morcom, 2016), regional specificity of feature modulation in the context of pattern separation specifically (as opposed to a domain-general contribution to memory processing; e.g., Stevenson et al., 2020) requires further investigation. Functional specialization of cognitive control regions is an area of active research and debate (e.g., Corbetta and Shulman, 2002; Badre and Nee, 2018), with some evidence suggesting that, within the lateral frontal cortex, caudal regions are more sensitive than rostral regions to stimulus features (Nee and D’Esposito, 2016; Nee and D’Esposito, 2017). Regional specificity, however, can be complicated by several factors, including age of the individual and task demands, that influence which regions are activated and the level of their activation (e.g., Grady, 2012). Uncovering the regional specificity of cognitive control regions that contribute to pattern separation is a question for future research. Nevertheless, we propose that feature-based attention and interference resolution by frontoparietal regions during pattern separation can regulate the degree of representational distinctiveness, and increase the neural representation of unique features, in sensory regions feeding into upstream MTL regions and the hippocampus (hence contributing to the perceptual pattern separation signal detected in these regions in some studies; e.g., Kim et al., 2009; Bowman et al., 2019; Klippenstein et al., 2020), and thus forms the basis of the filtered or ‘separated’ input signal reaching the hippocampus. Once this signal reaches extra-hippocampal MTL cortices, such as the PrC, domain-selective (i.e., object vs. spatial) interference resolution based on modulated features takes place before the final domain-general, hippocampally based pattern separation (Reagh and Yassa, 2014; Berron et al., 2018; Reagh et al., 2018).

In support of the proposal of pattern separation via attentionally based modulation of sensory regions, one study has demonstrated that lateral frontal and occipital regions show greater functional connectivity when correctly identifying lure objects as ‘new’ (i.e., engaging in successful pattern separation), relative to falsely endorsing them as ‘old’ (Wais et al., 2017). Furthermore, fMRI and ECoG studies have shown that encoding activity in lateral frontal regions temporally precedes activity in the hippocampus, and that this temporal dynamic is associated with successful detail-oriented memory formation (Long and Kahana, 2015; Cooper and Ritchey, 2020). Additionally, recent fMRI and electroencephalogram (EEG) work has illustrated that frontally mediated reactivation and early discrimination of low-level perceptual features in sensory regions during recall/processing of stimuli is associated with the ability to correctly discriminate those stimuli from similar lures (Bone et al., 2020; Herman et al., 2020). Finally, animal studies provide convincing evidence that (1) frontal feature-coding and input to the hippocampus drives memory recall, enabling high-fidelity retrieval (e.g., to distinguish between similar memory representations; Yadav et al., 2022), and (2) inactivation of the rodent frontal cortex and disruption of hippocampal input by blocking frontal–perirhinal communication, impairs performance on hippocampal-dependent memory tasks, including mnemonic discrimination of highly overlapping stimuli (Davies et al., 2017; Hernandez et al., 2017; Jayachandran et al., 2019; Johnson et al., 2021; see ‘Cross-species and developmental considerations’). Clearly, however, more human research is needed to establish a link between frontoparietal control function and sensory representations during pattern separation tasks, and importantly, between top-down modulated sensory input and hippocampal pattern separation and representations.

An important consideration in the CHiPS framework is how interference between overlapping stimuli is detected to engage frontoparietal control regions. One possibility is that novelty detection mechanisms that occur during early stages of stimulus processing signal the need for frontoparietal regions to increase attention to unique stimulus features and resolve potential interference. These novelty computations, which result from processing of familiar stimuli more ‘fluently’ than novel stimuli, have been shown to occur in several regions including perceptual and hippocampal regions (e.g., Knight, 1996; Czigler et al., 2004; Kim et al., 2009). For example, work on macaques has demonstrated stimulus-specific reductions in neuronal firing rates in regions such as the inferior temporal cortex in response to stimulus repetition (even with many intervening stimuli) approximately 150-ms post-stimulus onset (and as early as 70 ms in other regions; see Grill-Spector et al., 2006). Thus, perceptual and hippocampal regions likely carry out the mnemonic comparator process between overlapping stimuli required for pattern separation (based on stimulus-specific novelty computations) and provide feedback to frontoparietal control regions to modulate attention to shared/unique stimulus features. This enhances the processing of lure items and initiates the process of forming non-overlapping memory traces for these items (see bidirectional arrows in Figure 2). The extent to which early mnemonic comparator processes depend on perceptual or hippocampal regions may depend on factors such as mnemonic load, with the hippocampus playing a more significant role when an incoming stimulus is compared to a stored representation over longer delays (e.g., perceptual vs. mnemonic discrimination; see Weeden et al., 2014). Thus, early feedback signals from perceptual and/or hippocampal regions can engage interference resolution function in frontoparietal regions, which in turn modulates neural representations in sensory regions and regulates hippocampal input used for memory formation.

An alternative, but not mutually exclusive, possibility of how cross-region interactions support pattern separation is through direct interactions between cognitive control regions and the hippocampus, rather than indirectly via modulation of sensory regions. In this case, attentional modulation would directly bias hippocampal function and representations (after early novelty signals from perceptual and hippocampal regions), thus enhancing pattern separation in accordance with task demands (Figure 2). Consistent with this hypothesis, multiple studies have now illustrated that hippocampal activity patterns differ between distinct task-dependent goal states, and that these representations differ even when participants are viewing the same scene but are attending to different features of those scenes (e.g., Tambini and Davachi, 2013; Aly and Turk-Browne, 2016a; Aly and Turk-Browne, 2016b; Günseli and Aly, 2020; for a review see Aly and Turk-Browne, 2018). That is, representations or activity patterns in the hippocampus change in accordance with goal states or task demands and reflect the stimuli that are being attended to (see also Carr et al., 2013). Interestingly, one study demonstrated that hippocampal patterns represent different stimulus features of the very same objects in accordance with changing task goals (a critical function for the ability to distinguish between similar items with overlapping features), and that this effect is associated with functional connectivity between the hippocampus and a set of prefrontal regions, including the dorsolateral PFC (Mack et al., 2016; see Yadav et al., 2022 for similar evidence in animals). Moreover, converging evidence from fMRI and ECoG studies has demonstrated that the dorsolateral PFC exerts top-down inhibitory control on the hippocampus during encoding or retrieval of intrusive information, providing further support for task-dependent prefrontal–hippocampal regulation (Benoit and Anderson, 2012; Benoit et al., 2015; Oehrn et al., 2018; see also Malik et al., 2022). This regulation is possibly driven by the documented direct anatomical connections from the frontal cortex to the hippocampus (see Goldman-Rakic et al., 1984; Rajasethupathy et al., 2015; Malik et al., 2022 for evidence of such connections in non-human animals), or by fronto-hippocampal projections that traverse through intermediate structures, such as the anterior cingulate cortex and thalamus (see Anderson et al., 2016 for a review).

A few studies have provided evidence of attention-based hippocampal modulation during pattern separation tasks, suggesting a possible direct influence from cognitive control regions. For example, different studies have illustrated, based on both univariate and multivariate analyses, that in the Mnemonic Similarity Task, the hippocampus shows evidence of pattern separation for similar items only when task demands require a behavioral distinction to be made between similar and old items (Hashimoto et al., 2012; Lohnas et al., 2018). That is, hippocampal pattern separation was seen when items needed to be distinguished based on (fine-grain) perceptual, but not (coarse-grain) conceptual, features (e.g., a beach ball with slightly different features than a previously presented one would, respectively, be classified as ‘new’ and ‘old’). Similarly, another study demonstrated that hippocampal activation in response to graded changes in the similarity of lures to old items is influenced by encoding instructions or top-down demands (Motley and Kirwan, 2012). Particularly, in a continuous recognition task, hippocampal activation was better fit with a power function (i.e., the typical characterization of pattern separation) under intentional instructions that required participants to distinguish between the items based on fine-grain features, relative to incidental, category classification instructions. Further suggesting that cognitive control regions modulate the hippocampus during this task (continuous recognition task with intentional instructions), a recent study illustrated that a cognitive control dorsomedial frontal region showed functional connectivity with the hippocampus during task performance and displayed an activity pattern consistent with pattern separation (Nash et al., 2021). Finally, an anatomical study demonstrated that the integrity of the fornix, which connects the hippocampus to multiple regions including inferior prefrontal regions, is associated with behavioral performance on a pattern separation, but not recognition memory, task (Bennett et al., 2015). Although the fornix is typically considered to be a major output of the hippocampus, or hippocampal efferent, studies have demonstrated that it also contains afferent fibers feeding into the hippocampus (e.g., Schmahmann and Pandya, 2009). Thus, this finding provides anatomical support for the hypothesis that cognitive control regions might influence pattern separation that occurs within the hippocampus. Although these studies provide evidence of hippocampal modulation during pattern separation tasks, the question of whether these effects are indeed due to a direct influence from cognitive control regions or are mediated by signals relayed through sensory regions (see above) requires further investigation.

### Cross-species and developmental considerations

Thus far, we have reviewed evidence supporting the CHiPS framework in human studies. Yet, the question remains whether extra-hippocampal contribution to pattern separation has increased with evolution and is more evident in humans or whether similar processes occur in other species with less developed cortical function. In line with human studies, emerging evidence has indicated that indeed pattern separation both occurs outside the hippocampus and relies on frontal activity and input in rodents. For example, studies have shown that the olfactory bulb contributes to odor acuity in rodents by separating highly overlapping odors. In particular, similar to pattern separation processes reported in the DG, electrophysiological recordings from neural ensembles in the rodent olfactory bulb showed significant decorrelations in spike activity in response to highly overlapping odor mixtures (Barnes et al., 2008; Wilson, 2009; Sahay et al., 2011). Furthermore, the extent of perceptual pattern separation in the olfactory bulb was more recently shown to predict rodents’ ability to behaviorally discriminate between similar odors (i.e., greater pattern separation was correlated with faster odor discrimination learning; Gschwend et al., 2015).

In addition to odor perceptual pattern separation, recent work has shown that mnemonic discrimination in rodents, a process previously shown to rely on the DG (e.g., Johnson et al., 2019), also depends on frontal activity and input to the hippocampus (Johnson et al., 2021). In the study, rats performed an object-based mnemonic discrimination task, in which they were required to discriminate between a learned target object and lures with varying degrees of similarity to receive a reward. Rats with an inactive medial frontal cortex (i.e., blocked neural activity due to muscimol infusions) showed impaired mnemonic discrimination performance relative to controls, particularly when lures were highly similar to the target, demonstrating a pattern separation deficit. Similarly, in another study, inactivation of the medial frontal cortex was shown to impair pattern separation performance in a delayed nonmatch-to-location spatial task that required rats to discriminate the location of two closely presented stimuli for a food reward (Davies et al., 2017). These results suggest that the rodent frontal cortex is critical for pattern separation possibly through modulating and providing input to the hippocampus, given that it shows reciprocal connections with parahippocampal cortices that directly feed into the hippocampus (e.g., Delatour and Witter, 2002). Additionally and similar to work on humans, studies have illustrated that upstream MTL regions, such as the PrC, providing hippocampal input are critical for the perceptual and mnemonic discrimination of objects with high featural overlap across different animal species (e.g., Burke et al., 2011; Miranda et al., 2017; see also Burke et al., 2018 for evidence of how transection of perforant path input from the ErC to the DG impairs performance on the object-based mnemonic discrimination task). These findings are not surprising given the wealth of evidence highlighting the intimate relationship between the hippocampus and frontal activity/input in other types of memory tasks. For instance, blocking medial frontal activity or frontal–perirhinal communication that disrupts hippocampal input, has been shown to impair hippocampal-dependent object-place associative memory and temporal sequence memory (Hernandez et al., 2017; Jayachandran et al., 2019; see also Chung et al., 2021).

Finally, it is worth noting that in addition to animal studies, human development work suggests that pattern separation abilities in children and older adults, respectively, develop and decline in parallel with changes in prefrontal maturity/function and connectivity with the hippocampus during memory tasks (see Grady et al., 2003; Ofen et al., 2007; Ofen et al., 2012; Stark et al., 2013; Devitt and Schacter, 2016; Rollins and Cloude, 2018; Tang et al., 2018; Ngo et al., 2019; Gellersen et al., 2021; Weeks et al., 2020). This suggests that, in addition to the contribution of age-related hippocampal changes (e.g., Yassa et al., 2011a; Canada et al., 2019), developmental changes in extra-hippocampal processes may, at least partly, account for pattern separation performance across the lifespan. Future research should directly investigate the relationship between age-related changes in cognitive control function/hippocampal cortical input and pattern separation. In the case of older adults, given that pattern separation deficits are considered one of the defining features of aging, one potentially important outcome of the CHiPS framework is to examine the extent of the contribution of the distinct stages of pattern separation, and their interaction, to these deficits. Starting with the well-documented age-related cognitive control deficits (e.g., Amer et al., 2016), it will be important to investigate how reduced top-down modulation of downstream regions (and overrepresentation of irrelevant information/features) feeding into the hippocampus impacts hippocampal computations, representations, and memory structure (see Amer et al., 2022 for a discussion of changes in memory structure with old age). Additionally, studying interactions between age-related attentional or control deficits and structural and functional alterations of MTL regions, including the hippocampus (e.g., Yassa et al., 2011a; Reagh et al., 2018) will be critical for our understanding of how age-related changes in brain-wide function contribute to pattern separation deficits and mnemonic interference with old age. For instance, ‘noisy’ representations reaching the hippocampus due to age-related reductions in initial filtering mechanisms (as well as perceptual deficits) can place further demands on an already strained MTL system, reducing the likelihood of effective pattern separation. In sum, the discussed findings provide further support for the CHiPS framework and suggest that evidence of brain network contributions to behavioral pattern separation can be seen across species and possibly different stages of human development.

### A unique hippocampal role in pattern separation?

In the previous section, we discussed the potential influence of extra-hippocampal regions on hippocampal pattern separation through the CHiPS framework and mentioned that the hippocampus ultimately forms unique memory traces for overlapping stimuli based on the input it receives. This raises questions about the unique operations that hippocampal circuitry supports, which might include the amplification of the differences in similar inputs as well as their persistence in memory. Based on classic work suggesting that the DG amplifies small changes in its cortical input (e.g., O’Reilly and McClelland, 1994), it is possible that one mechanism by which the hippocampus forms separate memory traces is repelling overlapping hippocampal representations further apart, such that minor differences between similar stimuli are *exaggerated* in memory. While this operationalization of hippocampal function is not distinct from prior theories (see Hulbert and Norman, 2015), the CHiPS framework proposes that this function is initiated in, and modulated by, regions outside the hippocampus. That is, extra-hippocampal regions initiate pattern separation (transform similar inputs into less similar outputs) and transmit this initially separated signal to the hippocampus, biasing it toward pattern separation (see Bein et al., 2020a for evidence of how the hippocampus can be biased into ‘encoding’ or ‘retrieval’ states). Once the input signal reaches the hippocampus, it then importantly performs the fine tuning necessary to lay down unique memory traces by inducing ‘repulsions’ in its representations of the similar stimuli (i.e., already ‘separated’ similar inputs are transformed by a greater magnitude into even less similar and differentiated outputs). Specifically, by assigning dissimilar neural populations to highly similar stimuli or events (sparse coding), the hippocampus actively separates overlapping events by amplifying their differences in their memory representations. This is consistent with the interpretation that hippocampal pattern separation can be ‘arbitrary’ or even ‘unnecessary’ for already separated or orthogonalized input (Santoro, 2013). Indeed, several fMRI studies have demonstrated this effect by illustrating that over the course of learning overlapping stimuli, hippocampal representations of these stimuli are differentiated to the point of becoming *less* similar than non-overlapping stimuli (i.e., the representations are separated beyond simple orthogonalization; Hulbert and Norman, 2015; Favila et al., 2016; Chanales et al., 2017; Dimsdale-Zucker et al., 2018). A recent study demonstrated the behavioral effect of this repulsion by showing that long-term memories of similar stimuli are biased away from one another, such that they are remembered as being *more* different than they actually were (Chanales et al., 2021). Critical to the importance of the CHiPS framework, this effect was eliminated when task demands did not require differentiation of the similar stimuli, providing evidence that hippocampal pattern separation processes are modulated by top-down goals or processes. Finally, it is important to note that although there is evidence supporting the notion of representational repulsion in the hippocampus, this evidence is typically based on studies that use many training trials to study the overlapping stimuli, and critically, the same effect has also been reported in lateral frontal regions (Schlichting et al., 2015). Thus, whether this effect occurs for overlapping stimuli/events encountered in a single trial (or is particularly a form of separation that grows with learning), and whether this effect is truly unique to the hippocampus, are questions that require future investigation. It is possible that the initial pattern separation that emerges from extra-hippocampal and hippocampal operations during only a single trial may grow stronger with subsequent repetitions and learning. Finally, it is becoming increasingly clear that one of the fundamental contributions of the hippocampus to cognition occurs during offline states when recently formed neural ensembles can undergo repeated replay to strengthen those potential engrams in the hippocampus and in cortical regions (Wilson et al., 2006; Diba and Buzsáki, 2007; Tambini et al., 2010; Tambini and Davachi, 2019; Tambini and Davachi, 2013). Thus, it is important to consider the distinct contributions between online and offline contributions to pattern separation, and how the hippocampus might play a particularly significant role for the latter.

### Conclusion

Decades of research focusing on behavioral discrimination have emphasized the prominent role of the hippocampus in pattern separation, shifting focus away from critical extra-hippocampal contributions. This research priority is in need of re-evaluation in light of converging evidence from a wide range of studies suggesting that pattern separation encompasses a network of regions and is not restricted to the hippocampus. Based on such evidence, we propose in the CHiPS framework that cognitive control regions may contribute to pattern separation by modulating neural responses in sensory regions feeding into the hippocampus, thus regulating its cortical input, or through direct modulation of hippocampal function in accordance with task demands. Considering research illustrating that the hippocampus flexibly incorporates task goals and input from the rest of the brain to support various memory functions (e.g., Tambini and Davachi, 2013; Aly and Turk-Browne, 2018; Bein et al., 2020b; Brunec et al., 2020), it is no surprise that pattern separation would be similarly mediated by common neocortical–hippocampal interactions. The importance of the CHiPS framework is that it generates testable mechanistic questions with regard to how interactions between different regions may impact or support hippocampal pattern separation. For example, how does hippocampal pattern separation interact with different attentional demands, and does this effect vary as a function of activity in regions associated with cognitive control? How is activity in higher-order regions during mnemonic discrimination tasks related to differentiation of representations in the hippocampus? Are these differentiation effects mediated by representations (and interference resolution) in sensory regions feeding into the hippocampus? Finally, how do age-related changes in top-down control mechanisms contribute pattern separation deficits? To address these questions, further research with fine-grained temporal resolution will be necessary to determine how top-down control mechanisms impact distinct stages of pattern separation by targeting different brain regions.
